# Socio-demographic, pattern of presentation and management outcome of breast cancer in a semi-urban tertiary health institution

**DOI:** 10.11604/pamj.2020.36.363.17866

**Published:** 2020-08-28

**Authors:** Julius Gbenga Olaogun, John Adetunji Omotayo, Joshua Taye Ige, Abidemi Emmanuel Omonisi, Olusoga Olusola Akute, Olufunso Simisola Aduayi

**Affiliations:** 1 Department of Surgery, Ekiti State University, Ado-Ekiti, Nigeria,; 2 Department of Anatomic Pathology, Ekiti State University, Ado-Ekiti, Nigeria,; 3 Department of Radiology, Ekiti State University, Ado-Ekiti, Nigeria

**Keywords:** Breast cancer, clinical presentation, socio-demographics, treatment outcome

## Abstract

**Introduction:**

breast cancer is the most common malignancy in females worldwide and a major cause of cancer-related deaths in both developing and developed countries. The objective of this study was to determine the socio-demographics, pattern of presentation and management outcome of breast cancer patients.

**Methods:**

clinical records of confirmed breast cancer patients between January 2011 and December 2015 at the Ekiti State University Teaching Hospital, Ado-Ekiti, Nigeria were reviewed.

**Results:**

eighty two breast cancer patients were seen. Their ages ranged from 26-95 years (mean 48.9 ± 14.9 years, median 47.5 years). Eighty one (98.8%) were females and the majority (65.4%) were premenopausal. The peak age of incidence was in the 4^th^ decade. All patients presented with breast lump with mean duration of 9.49±6.1 months and size ranging from 2 to 16cm (mean 7.9±3.4 cm). Ten (12.2%) patients presented early, 61 (74.4%) were locally advanced while 11 (13.4%) had distant metastases. Fifty one (62.2%) patients had mastectomy. Only 38 (46.3%) patients completed six courses of chemotherapy. None had immunohistochemistry but they all routinely took tamoxifen. Only 4 (4.9%) had radiotherapy. Nineteen (23.2%) died within a year of presentation. The follow-up period ranged between 1 and 44 months (mean, 10.3 months). Thirty one (37.8%), 19 (23.2%) and 8 (9.8%) patients were seen during the first, second and third year of follow up respectively.

**Conclusion:**

breast cancer mostly affects young premenopausal women presenting in advanced stage in our setting. The generally poor outcome is not unconnected with late presentation and inadequate diagnostic and treatment facilities.

## Introduction

Breast cancer is the most common malignancy in females worldwide and a major cause of cancer-related deaths in both developing and developed countries. Over 1.4 million women are diagnosed of breast cancer every year [1, 2]. About 32% of all cancer cases and 18% of all cancer deaths in women are reportedly due to breast cancer [3]. There has been a steady increase in the incidence of breast cancer in Nigeria from 15.3 per 100,000 in 1976 to 33.6 per 100,000 in 1992 to 52.0 and 64.6 per 100,000 in 2012 in Ibadan and Abuja respectively [4, 5]. This may not be unconnected with the recent increase in women´s level of awareness and knowledge of breast cancer disease making them to seek medical intervention in many health institutions.

The hallmark of breast cancer disease in Nigeria, like other developing African countries, is late presentation and this among other factors is majorly responsible for poor treatment outcome in terms of survival. In this advanced stage, there is less than 10% five-year survival rate in Nigeria [4, 6-8]. The reverse is the case in the United States where the overall 5-year survival is more than 90% and still continues to rise even with the rising incidence of breast cancer [9]. This study was carried out to evaluate the socio-demographics, pattern of presentation and management outcome of breast cancer patients in Ekiti State University Teaching Hospital, Ado-Ekiti, Nigeria.

## Methods

This was a descriptive retrospective study carried out at the Ekiti State University Teaching Hospital (EKSUTH), Ado-Ekiti. The hospital is a 350-bed tertiary institution located in a semi-urban area of Ekiti and serves as a referral center for the primary and secondary health facilities in Ekiti State and other neighbouring States like Osun, Kwara, Ondo, Kogi, and Edo in Southern Nigeria. This study evaluated all patients with histologically confirmed breast cancer who were managed in our hospital between January 2011 and December 2015 and who received at least one form of treatment. All cases with benign breast lesions, suspected breast cancers that were not histologically confirmed and those who defaulted after the first clinic presentation were excluded from the study. The authors obtained ethical clearance for this study from the EKSUTH Ethics and Research Committee.

During this study period, we reviewed surgical outpatient clinic, emergency department and theatre registers to extract the names and hospital numbers of patients managed for breast cancer. The list generated was used to retrieve patients´ case notes that met the inclusion criteria from the medical records department. The information sought from the case notes using a proforma designed for the study included: patients´ socio-demographics, clinical stage at presentation, obstetrics/ gynaecology history, diagnostic methods, treatment modalities and outcome in terms of survival and mortality. All patents routinely had abdomino-pelvic scan, plain chest radiograph and histological diagnosis within 6 weeks of presentation. According to the American Joint Committee on Cancer (AJCC 2002) TNM stage groupings, the patients were categorized into early stage (stages 0, 1, IIA and IIB), locally advanced (IIIA-C) and metastatic (stage IV). The data generated were entered into a spread sheet and analyzed using the statistical package for social sciences (SPSS) version 21.0. (IBM incorporated).

## Results

**Figure 1:**
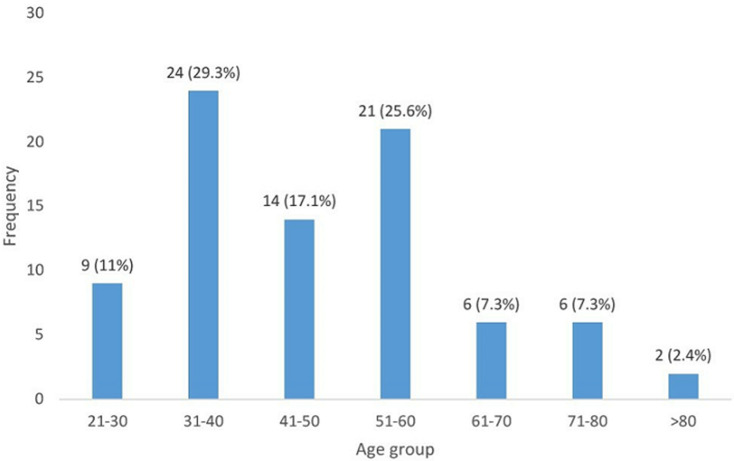
age distribution of the patients

A total number of 654 patients with breast lesions were seen during the period of which 82 (12.5%) were confirmed breast cancer by either needle or open surgical biopsy. Their ages ranged between 26 and 95 years (mean, 48.9 ± 14.9 years). The median age was 47.5 years. The age distribution of the patients is shown in Figure 1 below. The peak age of incidence was seen in the fourth decade followed by 6^th^ decade. The socio-demographic profiles of the patients are shown in Table 1. Eighty one (98.8%) of the patients were females, 53 (64.6%) at least had secondary education, 63 (76.8%) were multiparous while 53 (64.6%) were premenopausal.

**Table 1:** socio-demographic profiles of patients

Socio-demographics	Frequency (%)
**Sex**	
Female	81 (98.8)
Male	1 (1.2)
**Educational status**	
None	22 (26.8)
Primary	7 (8.5)
secondary	17 (20.7)
Tertiary	36 43.9)
**Marital status**	
Single	4 (4.9)
Married	64 (78.0)
Divorced/separated	5 (6.1)
Widowed	9 (11.0)
**Parity**	
0	6 (7.3)
1	13 (15.9)
2-4	42 (51.2)
>4	21 (25.6)
**Menopausal status**	
Pre-menopausal	53 (64.6)
Post-menopausal	28 (34.2)
Not applicable	1 (1.2)
**Religion**	
Christianity	79 (96.3)
Islam	3 (3.7)
**Occupation**	
None	12 (14.6)
Students	2 (2.4)
Artisans	4 (4.9)
Traders	26 (31.7)
Farmers	3 (3.7)
Teachers	19 (23.2)
Civil servants	12 (14.6)
Nurse/health assistants	4 (4.9)

Table 2 shows the symptoms at presentation. All the patients (100%) had history of breast lump. The duration of breast swelling ranged from 1 to 36 months with a mean duration of 9.49 ± 6.1 months. The mean lump size was 7.9 ± 3.4 cm (range, 2-16cm). The left breast was affected in 44 (53.7%), right breast in 36 (43.9%) while 2 (2.4%) patients had bilateral breast cancer. The most common histological diagnosis of the breast tumour was invasive ductal carcinoma (91.5%). Other histological variants are seen in Table 3. According to the American Joint Committee on Cancer (AJCC) Stage Groupings (Table 4), only 10 (12.2%) patients presented early, the vast majority 61 (74.4%) of the patients presented with locally advanced disease while 11 (13.4%) had distant metastases involving the pleura, lung, liver, bone and brain.

**Table 2:** symptoms at presentation

Symptoms^*^	Frequency (%)
Breast lump	82 (100)
Axillary lump	53 (64.6)
Nipple deformity	38 (46.3)
Pain	37 (45.1)
Ulceration	21 (25.6)
Itching	18 (21.9)
Abnormal nipple discharge	9 (11.0)
Features of metastasis	11 (13.4)

* Some patients had more than one symptom at presentation

**Table 3:** histologic types of the breast cancer

Histology	Frequency (%)
Invasive ductal carcinoma	75 (91.5)
Papillary carcinoma	2 (2.4)
Malignant melanoma	1 (1.2)
Squamous cell carcinoma	1 (1.2)
Tubular carcinoma	1 (1.2)
Angiosarcoma	1 (1.2)
Fibrosarcoma	1 (1.2)
**Total**	100.0

**Table 4:** AJCC group stage of patients

Stage	Frequency (%)
Stage 0	0 (0.0)
Stage 1	1 (1.2)
Stage II	
A	1 (1.2)
B	8 (9.8)
Stage III	
A	12 (14.6)
B	45 (54.9)
C	4 (4.9)
Stage IV	11 (13.4)
**Total**	82 (100.0)

Modified radical mastectomy was performed on 45 (54.9%) patients while 6 (7.3%) had simple mastectomy. Thirty six (70.6%) received neo-adjuvant chemotherapy ranging from 1 to 6 courses while the rest only had adjuvant chemotherapy. All patients received at least a dose of anthracycline based chemotherapy regimen (cyclophosphamide, doxorubicin/epirubicin and 5-fluorouracil) but only 36 (43.9%) completed six courses while the rest were not regular on medications mostly for financial constraints. The immunohistochemistry of the breast tissue could not be determined for lack of facility. However, all the patients were routinely placed on tamoxifen but none received biological therapy. Of the 26 patients referred for adjuvant radiotherapy, only 4 (15.4%) had it done. Nineteen (23.2%) died within a year of presentation. The follow-up period ranged between 1 and 44 months (mean, 10.3 months). Thirty one (37.8%), 19 (23.2%) and 8 (9.8%) patients were seen during the first, second and third year of follow up respectively.

## Discussion

There has been an increase in the incidence of breast cancer worldwide and it is presently the most common female malignancy in Nigeria. In our study, 12.5% of patients seen had breast cancer. Breast cancer affects younger population of women with a mean age of 48 years. This was similar to studies from different parts of Nigeria and Africa [10-12]. In a study by Bowen *et al*. [13] in UK, the British black women also presented younger at a median age of 46 years than white patients at median age of 67 years. The peak incidence was in the 4^th^ decade closely followed by 6^th^ decade in what looks like bimodal distribution of the disease. Most breast cancers are found in the females. Our finding of 1.2% of breast cancer in the male conforms to what has been documented by many studies in the literature. About two-thirds of the women were premenopausal or perimenopausal at presentation. This finding was similar to 66.7% and 69% reported by Adesunkanmi *et al*. [10] and Anyanwu [14] in South-Western and South-Eastern parts of Nigeria respectively. This further lends credence to the fact that breast cancer affects younger population of women in African countries. However, this was contrary to what obtains in Europe and America where the majority of the women are postmenopausal [15-17]. Nulliparity or low parity increases the risk of developing breast cancer. Although this study did not seek to find the risk factors for breast cancer in our locality, we found that the majority (76.8%) of our patients were multiparous and this was similar to previously published studies [10, 14].

Most of our patients were Christians. As much as this does not have any bearing on the aetiopathogenesis of this disease, it sometimes negatively impacts presentation to the hospital. A significant number of these people live in self-denial and would want to seek help from their spiritual leaders thereby delaying presentation. The most common symptom at presentation was breast lump. All our patients presented with breast lump with a mean duration of 9.5 months and lump size of 7.9 cm and the majority (64.6%) were having clinically palpable axillary lymph nodes. Rambau *et al*. [12] in their study reported that the tumor size greater than 6 cm in diameter and presence of necrosis within the tumor was significantly associated with high rate of lymph node metastasis. A quarter of our patients also presented with breast ulceration. All these are in keeping with late presentation which is the hallmark of breast cancer presentation in Nigeria and other developing countries. Since breast lump is the most common symptom, this should be emphasized during health awareness programs for women and the health professionals should play a vital role in educating women during their hospital visits even for other health problems. It is said that more than 90% of cases of breast cancers are detected by women themselves, stressing the importance of breast self - examination for early detection and treatment of cancer [10, 18, 19].

According to AJCC, the majority, 72 (87.8%) of patients, presented in stages III and IV disease. Studies from different parts of Nigeria and most developing countries showed that most patients presented in stages III and IV [10, 12, 14, 20-22]. However, the percentages of patients who presented with metastatic disease in the studies by Ntekim *et al*. [20] and Agbo *et al*. [21] were abysmally higher than our findings. Different reasons have been adduced to late presentation in Africans among which are illiteracy or low education, fear of diagnosis, ignorance, denial, belief in unorthodox therapies and faith healing [23]. The predominant histological type of invasive ductal carcinoma in our setting was similar to what have been reported in different studies [12, 14, 20, 21] The majority (64.6%) of patients in this setting had at least a secondary school education but was not found to have positively impacted the presentation. Adisa *et al*. [24] in Ile-Ife and Ntekim *et al*. [20] in Ibadan reported higher rates of 73.8% and 85% of at least a secondary formal education in their patients but the former noted that the high educational level however did not influence the duration of symptoms before presentation as only (34.7%) presented within 3 months of noticing breast lump. Ideally, one would expect good education to translate to patients´ better awareness and knowledge of the symptomatology of breast cancer. The paradox of a highly educated individual presenting late to the hospital may not likely change when there is lack of required funds for treatment and patients are unwilling to have a mastectomy [25].

The management of breast cancer is multimodal and this could be very challenging to physicians in the developing countries considering the advanced stage at presentation and the inadequacy of the diagnostic and treatment facilities. The choice of therapy is more often than not dictated by the local availability of resources [26]. The majority (54.9%) of our patients had modified radical mastectomy. This still remains the standard surgical operation most frequently performed for operable breast cancer in most parts of the world. Different surgical rates have been reported across Nigeria and different parts of Africa [11, 19, 25, 27]. The role of surgery for the loco-regional clearance cannot be over-emphasized as it represents the most popular option when there is limited infrastructure and access to chemotherapy and radiotherapy [28]. None of the patients in our setting had breast-conserving surgery because they mostly presented in advanced stages. Even in early breast cancer, this surgical option will remain largely impracticable due to limited availability of radiotherapy facilities across Nigeria and for the fact that most patients are usually lost to follow-up [10, 21]. About two-fifth completed six courses of anthracycline-based chemotherapy and there were high rates of interruptions and abrupt termination of cycles. This could be due to financial constraint as most patients in our settings are poor. Taxane based regimens were not used either because of non-availability or high cost. In a setting where late presentation is the norm, chemotherapy becomes an ideal for both treatment and palliation. The government has a vital role to play in ensuring that drugs are highly subsidized, readily available and affordable to guarantee a standard of care and better outcome.

All patients in this review routinely had tamoxifen. It is still a common practice to give anti-oestrogen blindly in Nigeria without recourse to oestrogen/progesterone receptor (ER/PR) status. This trend was also reported by Scherber *et al*. [29] in Ghana. This might have been done as a cover up for adequate treatment of patients in the face of limited diagnostic facilities. However, this falls short of international best practices of evidenced based medicine and should be discouraged. For the same reasons of lack of facility for determination of tumour markers and high cost, none of our patients benefitted from targeted therapy. Only 4 (4.9%) patients benefitted from radiotherapy. This may not be unconnected with few facilities often unevenly distributed in private or tertiary hospitals thereby limiting accessibility to many segments of the population, prohibitive cost, long waiting times and inconsistency of services due to high patients´ load at those centres. Two of our patients were given three months appointments and could not have radiotherapy on their scheduled dates as a result of faulty machine. The death of almost a quarter of patients within a year of presentation is a pointer to the fact that advanced stage of breast cancer is associated with poor prognostic outcome mostly reported in the low and middle income countries [10, 24, 30]. The follow-up is also generally poor among Nigerian patients with breast cancer [10, 21, 25]. Only 8 (9.8%) patients were seen in the third year of follow-up in this study. Considering the late presentation and numerous barriers to adequate treatments, it is not unlikely that the majority of these patients could have died. Various interventions and strategies to improve follow-up should be instituted during the initial contact with the patients.

## Conclusion

Breast cancer is seen in the young premenopausal women usually presenting in the advanced stage of the disease in our locality. The generally poor outcome is not unconnected with late presentation, inadequate diagnostic facilities and various treatment barriers. There is a need for more awareness to encourage early presentation and provision of adequate diagnostic and treatment facilities for improved outcome.

### What is known about this topic

- The hallmark of breast cancer disease in Nigeria, like other developing African countries, is late presentation when patients hardly benefit from any form of therapy;
- Advanced stage of presentation is what is majorly responsible for poor treatment outcome in terms of survival.

### What this study adds

- This study highlights the bimodal distribution of breast cancer disease in our setting with peaks at the 4^th^ and 6^th^ decades;
- High educational level does not necessarily translate to patients’ better awareness and knowledge of the symptomatology of breast cancer; the paradox of a highly educated individual presenting late to the hospital may likely persist;
- The generally poor outcome is not only due to late presentation but inadequate diagnostic and treatment barriers.

## References

[1] Siegel R, Naishadham D, Jemal A (2012). Cancer statistics. CA: A Cancer Journal for Clinicians.

[2] Ferlay J, Shin HR, Bray F, Forman D, Mathers C, Parkin DM (2010). Estimates of worldwide burden of cancer in 2008: GLOBOCAN 2008. Int J Cancer.

[3] Ahmad M (2003). Risk factors for breast cancer among women attending breast clinic in University Malaya Medical Centre Kuala Lumpur. NCD Malaysia.

[4] Adebamowo CA, Ajayi OO (2000). Breast cancer in Nigeria. West Afr J Med.

[5] Jedy-Agba E, Curado MP, Ogunbiyi O, Oga E, Fabowale T, Igbinoba F (2012). Cancer incidence in Nigeria: a report from population-based cancer registries. Cancer Epidemiol.

[6] Afolayan EAO, Ibrahim OOK, Ayilara GT (2012). Cancer Patterns in Ilorin: an Analysis of Ilorin Cancer Registry Statistics. The Tropical Journal of Health Sciences.

[7] Anyanwu SNC (2008). Temporal trends in breast cancer presentation in the third world. Journal of Experimental & Clinical Cancer Research.

[8] Okobia MN, Osime U (2001). Clinicopathological study of carcinoma of the breast in Benin City. Afr J Reprod Health.

[9] Ban KA, Godellas CV (2014). Epidemiology of breast cancer. Surg Oncol Clin N Am.

[10] Adesunkanmi ARK,Lawal OO, Adelusola KA, Durosimi MA (2006). The severity, outcome and challenges of breast cancer in Nigeria. Breast.

[11] Rahman GA, Olatoke SA, Agodirin SO, Adeniji KA (2014). Socio-demographic and clinical profile of immuno-histochemically confirmed breast cancer in a resource limited country. The Pan African Medical Journal.

[12] Rambau PF, Chalya PL, Manyama MM, Jackson KJ (2011). Pathological features of Breast Cancer seen in Northwestern Tanzania: A nine years retrospective study. BMC Res.

[13] Bowen RL, Duffy SW, Ryan DA, Hart IR, Jones JL (2008). Early onset of breast cancer in a group of British black women. Br J Cancer.

[14] Anyanwu SN (2000). Breast cancer in eastern Nigeria: a ten year review. West Afr J Med.

[15] Cancer Registry Finnish (2011). Cancer stat fact sheets, Helsinki, Finland: Institute for Statistical and Epidemiological Cancer Research.

[16] Registry of Norway Cancer (2011). Cancer in Norway: Cancer in Norway 2009 Cancer incidence, mortality, survival and prevalence in Norway. Oslo, Norway, Cancer Registry of Norway.

[17] Atlanta: American Cancer Society (2013). Breast Cancer Facts and Figures.

[18] Parkin DM, Muir CS, Whelan SL (1992). Cancer incidence in five continents. ARC Scientific Publication No. 120 IARC Lyon.

[19] Nguefack CT, Biwole ME, Massom A, Jacques Tsingaing Kamgaing, Theophile Nana Njamen, EKANE GH (2012). Epidemiology and surgical management of breast cancer in gynecological department of Douala General Hospital. Pan Afr Med J.

[20] Ntekim A, Nufu FT, Campbell OB (2009). Breast cancer in young women in Ibadan, Nigeria. Afr Health Sci.

[21] Agbo PS, Khalid A, Oboirien M (2014). Clinical presentation, prevalence and management of breast cancer in Sokoto, Nigeria. J Women´s Health Care.

[22] Ohene-Yeboah M, Adjei E (2012). Breast cancer in Kumasi, Ghana. Ghana Med J.

[23] Sharma K, Costas A, Shulman LN, Meara JG (2012). A systematic review of barriers to breast cancer care in developing countries resulting in delayed patient presentation. J Oncol.

[24] Adisa AO, Arowolo OA, Akinkuolie AA, Titiloye NA, Alatise OI, Lawa OO (2011). Metastatic breast cancer in a Nigerian tertiary hospital. Afr Health Sci.

[25] Ogundiran TO, Ayandipo OO, Ademola AF, Adebamowo CA (2013). Mastectomy for management of breast cancer in Ibadan, Nigeria. BMC Surg.

[26] Sutter SA, Slinker A, Balumuka DD, Mitchell KB (2016). Surgical Management of breast cancer in Africa: a continent-wide review of intervention practices, barriers to care, and adjuvant therapy. J Glob Oncol.

[27] Tesfamariam A, Gebremichael A, Mufunda J (2013). Breast cancer clinicopathological presentation, gravity and challenges in Eritrea, East Africa: management practice in a resource-poor setting. S Afr Med J.

[28] Kantelhardt EJ, Cubasch H, Hanson C (2015). Taking on breast cancer in East Africa: global challenges in breast cancer. Curr Opin Obstet Gynecol.

[29] Scherber S, Soliman AS, Awuah B, Ernest Osei-Bonsu, Ernest Adjei, Frank Abantanga (2014). Characterizing breast cancer treatment pathways in Kumasi, Ghana from onset of symptoms to final outcome: Outlook towards cancer control. Breast Dis.

[30] Basro S, Apffelstaedt JP (2010). Breast cancer in young women in a limited-resource environment. World J Surg.

